# Data Science in Healthcare: COVID-19 and Beyond

**DOI:** 10.3390/ijerph19063499

**Published:** 2022-03-16

**Authors:** Tim Hulsen

**Affiliations:** Department of Hospital Services & Informatics, Philips Research, 5656AE Eindhoven, The Netherlands; tim.hulsen@philips.com

Data science is an interdisciplinary field that applies numerous techniques, such as machine learning (ML), neural networks (NN) and artificial intelligence (AI), to create value, based on extracting knowledge and insights from available ‘big’ data [1]. The recent advances in data science and AI have had a major impact on healthcare already, as can be seen in the recent biomedical literature [2]. Improved sharing and analysis of medical data results in earlier and better diagnoses, and more patient-tailored treatments. This increased data sharing, in combination with advances in health data management, works hand-in-hand with trends such as increased patient-centricity (with shared decision making), self-care (e.g., using wearables), and integrated healthcare delivery. Using data science and AI, researchers can deliver new approaches to merge, analyze, and process complex data and gain more actionable insights, understanding, and knowledge at the individual and population level [3]. AI can be applied in all three major areas of early detection and diagnosis, treatment, as well as outcome prediction and prognosis evaluation [4]. ML algorithms can make predictions on how a disease will develop or respond to treatment, deep learning algorithms can find malignant tumors in magnetic resonance (MR) images and digital pathology images, and natural language-processing (NLP) algorithms can analyze unstructured documents with high speed and accuracy. These are just a few examples of what data science can do. This Special Issue focuses on how data science and AI are used in healthcare, and on related topics such as data sharing and data management. Since this Special Issue contains papers from 2020 to 2022, naturally there are a few papers about the COVID-19 pandemic: one on the determination of potential risk factors for the case fatality rate, one on the analysis of Arabic Twitter data to detect government pandemic measures and public concerns, and one on an enhanced sentinel surveillance system for outbreak prediction. There are also papers about data-sharing initiatives, depression treatment, the relationship between depression and metabolic status, cardiac thoracic pain, hand-foot-and-mouth disease infection, arteriovenous fistula (AVF) failure, chronic kidney disease (CKD) and breast cancer diagnosis.

“Coronavirus Disease 2019 (COVID-19): A Modeling Study of Factors Driving Variation in Case Fatality Rate by Country” by Pan et al. [5], “COVID-19: Detecting Government Pandemic Measures and Public Concerns from Twitter Arabic Data using Distributed Machine Learning” by Alomari et al. [6] and “Enhanced Sentinel Surveillance System for COVID-19 Outbreak Prediction in a Large European Dialysis Clinics Network” by Bellocchio et al. [7] all present research around the COVID-19 pandemic. Pan et al. [5] identified 24 potential risk factors driving variation in SARS-CoV-2 case fatality rate (CFR). Their model predicted an increased CFR for countries that waited over 14 days to implement social distancing interventions after the 100th reported case. Smoking prevalence and the percentage population over the age of 70 years were also associated with higher CFR. Hospital beds per 1000 and CT scanners per million were identified as possible protective factors associated with decreased CFR. Alomari et al. [6] proposes a software tool comprising a collection of unsupervised Latent Dirichlet Allocation (LDA) ML and other methods for the analysis of Twitter data in Arabic with the aim to detect government pandemic measures and public concerns during the COVID-19 pandemic. Using the tool, they collected a dataset comprising 14 million tweets from the Kingdom of Saudi Arabia (KSA) for the period 1 February to 1 June 2020. They detected 15 government pandemic measures and public concerns, and six macro-concerns (economic sustainability, social sustainability, etc.), and formulated their information-structural, temporal, and spatio-temporal relationships. Bellocchio et al. [7] present a sentinel surveillance system supported by an ML prediction model, whereby the occurrence of COVID-19 cases in a clinic propagates distance-weighted risk estimates to adjacent dialysis units. The system allows for a prompt risk assessment and a timely response to the challenges posed by the COVID-19 epidemic throughout Fresenius Medical Care (FMC) European dialysis clinics.

“Sharing Is Caring-Data Sharing Initiatives in Healthcare” by Hulsen [8] shows an analysis of the current literature around data sharing, and discusses five aspects of data sharing in the medical domain, namely publisher requirements, data ownership, growing support for data sharing, data sharing initiatives and how the use of federated data might be a solution. With federated data, there is no need for a centralized source database (with all its privacy issues), because the algorithm is brought to the data instead of the other way around. The author also discusses some potential future developments around data sharing, such as medical crowdsourcing and data generalists.

“Digital Training for Non-Specialist Health Workers to Deliver a Brief Psychological Treatment for Depression in Primary Care in India: Findings From a Randomized Pilot Study” by Muke et al. [9] evaluates the feasibility and acceptability of a digital program for training non-specialist health workers to deliver a brief psychological treatment for depression. This study, performed in Sehore (a rural district in Madhya Pradesh, India) adds to mounting efforts aimed at leveraging digital technology to increase the availability of evidence-based mental health services in low-resource primary care settings in.

“Association of Metabolically Healthy Obesity and Future Depression; Using National Health Insurance System Data in Korea from 2009–2017” by Seo et al. [10] investigates if depression and metabolic status are relevant by classifying them into the following four categories by their metabolic status and body mass index: (1) metabolically healthy non-obese (MHN); (2) metabolically healthy obese (MHO); (3) metabolically unhealthy non-obese (MUN); and (4) metabolically unhealthy obese (MUO). Their results show that the MHN ratio in women is higher than in men. In both men and women, depression incidence was the highest among MUO participants. In female participants, MHO is also related to a higher risk of depressive symptoms. This indicates that MHO is not an entirely benign condition in relation to depression in women. Therefore, reducing the number of metabolic syndrome and obesity patients in Korea will likely reduce the incidence of depression.

“Assessment of Thoracic Pain Using Machine Learning: A Case Study from Baja California, Mexico” by Rojas-Mendizabal et al. [11] aims to determine the correlated variables with thoracic pain of cardiac origin. Their analysis of 258 geriatric patients from Medical Norte Hospital in Tijuana (Baja California, Mexico) uses two ML techniques, i.e., tree classification and cross-validation. Their results suggest that among the main factors related to cardiac thoracic pain are dyslipidemia, chronic kidney failure, hypertension, diabetes, smoking habits, and troponin levels at the time of admission.

“Optimized Neural Network Based on Genetic Algorithm to Construct Hand-Foot-and-Mouth Disease Prediction and Early-Warning Model” by Lin et al. [12] discusses the high number of recent infections of hand-foot-and-mouth disease (HFMD). Previous research on the prevalence of HFMD mainly predicts the number of future cases based on the number of historical cases in various places, and the influence of many related factors that affect the prevalence of this disease is ignored. Existing early-warning research of HFMD mainly uses direct case report, which uses statistical methods in time and space to provide early-warnings of outbreaks separately. It leads to a high error rate and low confidence in the early-warning results. This paper uses ML methods to establish an HFMD epidemic prediction model with a high accuracy. Both incidence data and environmental (mostly weather) data are used.

“Development and Validation of a Machine Learning Model Predicting Arteriovenous Fistula Failure in a Large Network of Dialysis Clinics” by Ricardo et al. [13] derived and validated an arteriovenous fistula failure model (AVF-FM) based on ML. The model was trained in the derivation set (70% of initial cohort) by exploiting the information routinely collected in the Nephrocare European Clinical Database (EuCliD; 13,369 patients). Model performance was tested by concordance statistic and calibration charts in the remaining 30% of records. Feature importance was computed using the SHapley Additive exPlanations (SHAP) method. The model achieved good discrimination and calibration properties by combining routinely collected clinical and sensor data, requiring no additional effort by healthcare staff. Therefore, it can potentially facilitate risk-based personalization of AVF surveillance strategies.

In “Validation of a Novel Predictive Algorithm for Kidney Failure in Patients Suffering from Chronic Kidney Disease: The Prognostic Reasoning System for Chronic Kidney Disease (PROGRES-CKD)” by Ricardo et al. [14] a novel algorithm predicting end-stage kidney disease (ESKD) is described, named PROGRES-CKD. This Naïve-Bayes classifier accurately predicts kidney failure onset among chronic kidney disease (CKD) patients. Contrary to equation-based scores, PROGRES-CKD extends to patients with incomplete data and allows for the explicit assessment of prediction robustness in case of missing values. The algorithm may efficiently assist physicians’ prognostic reasoning in real-life applications.

Finally, Rasool et al. [15] discuss in “Improved Machine Learning-based Predictive Models for Breast Cancer Diagnosis” four different predictive models to improve breast-cancer diagnostic accuracy, as well as data exploratory techniques (DET) such as feature distribution, correlation, elimination and hyperparameter optimization. The Wisconsin Diagnostic Breast Cancer (WDBC) and Breast Cancer Coimbra Dataset (BCCD) datasets were used as input. They report a significant improvement in the models’ diagnostic capability with their DET. Therefore, the techniques can help to improve breast cancer diagnosis.

The manuscripts in this Special Issue give us only a brief overview of the wide use of data science in healthcare, and offer a glimpse into the future, where even faster computers and more advanced AI algorithms will make many more applications possible. For example, whereas many AI algorithms only use data from specific data types, this can be expanded to a combination of a wide range of patient-related (structured or unstructured) data, including clinical data, imaging data, digital pathology data, genomics data, data from wearables, and much more, to optimize the result for the patient. AI systems will not replace clinicians on a large scale, but rather will support their care for patients [16]. For example, AI can also be used to optimize the workflow in the hospital, or to create intelligent chatbots to help patients while reducing the workload for the clinicians. Furthermore, AI algorithms created in these times of COVID-19 might be of good use when managing similar pandemics in the future. It is probably safe to say that in ten years from now, there will not be a ‘Data Science in Healthcare’ Special Issue, because by that time almost everything in healthcare will be influenced by data science.

## References

[1] Hulsen T., Jamuar S.S., Moody A.R., Karnes J.H., Varga O., Hedensted S., Spreafico R., Hafler D.A., McKinney E.F. (2019). From Big Data to Precision Medicine. Front. Med..

[2] Hulsen T. (2021). Literature analysis of artificial intelligence in biomedicine. Pharm. Res. Pers. Med..

[3] Hulsen T., Moustafa A.A. (2021). Challenges and solutions for big data in personalized healthcare. Big Data in Psychiatry & Neurology.

[4] Jiang F., Jiang Y., Zhi H., Dong Y., Li H., Ma S., Wang Y., Dong Q., Shen H., Wang Y. (2017). Artificial intelligence in healthcare: Past, present and future. Stroke Vasc. Neurol..

[5] Pan J., St Pierre J.M., Pickering T.A., Demirjian N.L., Fields B.K.K., Desai B., Gholamrezanezhad A. (2020). Coronavirus Disease 2019 (COVID-19): A Modeling Study of Factors Driving Variation in Case Fatality Rate by Country. Int. J. Environ. Res. Public Health.

[6] Alomari E., Katib I., Albeshri A., Mehmood R. (2021). COVID-19: Detecting Government Pandemic Measures and Public Concerns from Twitter Arabic Data Using Distributed Machine Learning. Int. J. Environ. Res. Public Health.

[7] Bellocchio F., Carioni P., Lonati C., Garbelli M., Martínez-Martínez F., Stuard S., Neri L. (2021). Enhanced Sentinel Surveillance System for COVID-19 Outbreak Prediction in a Large European Dialysis Clinics Network. Int. J. Environ. Res. Public Health.

[8] Hulsen T. (2020). Sharing Is Caring-Data Sharing Initiatives in Healthcare. Int. J. Environ. Res. Public Health.

[9] Muke S.S., Tugnawat D., Joshi U., Anand A., Khan A., Shrivastava R., Singh A., Restivo J.L., Bhan A., Patel V. (2020). Digital Training for Non-Specialist Health Workers to Deliver a Brief Psychological Treatment for Depression in Primary Care in India: Findings from a Randomized Pilot Study. Int. J. Environ. Res. Public Health.

[10] Seo Y., Lee S., Ahn J.S., Min S., Kim M.H., Kim J.Y., Kang D.R., Hwang S., Vicheka P., Lee J. (2020). Association of Metabolically Healthy Obesity and Future Depression: Using National Health Insurance System Data in Korea from 2009–2017. Int. J. Environ. Res. Public Health.

[11] Rojas-Mendizabal V., Castillo-Olea C., Gómez-Siono A., Zuñiga C. (2021). Assessment of Thoracic Pain Using Machine Learning: A Case Study from Baja California, Mexico. Int. J. Environ. Res. Public Health.

[12] Lin X., Wang X., Wang Y., Du X., Jin L., Wan M., Ge H., Yang X. (2021). Optimized Neural Network Based on Genetic Algorithm to Construct Hand-Foot-and-Mouth Disease Prediction and Early-Warning Model. Int. J. Environ. Res. Public Health.

[13] Peralta R., Garbelli M., Bellocchio F., Ponce P., Stuard S., Lodigiani M., Fazendeiro Matos J., Ribeiro R., Nikam M., Botler M. (2021). Development and Validation of a Machine Learning Model Predicting Arteriovenous Fistula Failure in a Large Network of Dialysis Clinics. Int. J. Environ. Res. Public Health.

[14] Bellocchio F., Lonati C., Titapiccolo J., Nadal J., Meiselbach H., Schmid M., Baerthlein B., Tschulena U., Schneider M., Schultheiss U.T. (2021). Validation of a novel predictive algorithm for kidney failure in patients suffering from chronic kidney disease: The Prognostic Reasoning System for Chronic Kidney Disease (PROGRES-CKD). Int. J. Environ. Res. Public Health.

[15] Rasool A., Bunterngchit C., Tiejian L., Islam M.R., Qu Q., Jiang Q. (2022). Improved Machine Learning-Based Predictive Models for Breast Cancer Diagnosis. Int. J. Environ. Res. Public Health.

[16] Davenport T., Kalakota R. (2019). The potential for artificial intelligence in healthcare. Future Healthc. J..

